# Injection of I_K1_ through dynamic clamp can make all the difference in patch-clamp studies on hiPSC-derived cardiomyocytes

**DOI:** 10.3389/fphys.2023.1326160

**Published:** 2023-12-12

**Authors:** Arie O. Verkerk, Ronald Wilders

**Affiliations:** ^1^ Department of Medical Biology, Amsterdam Cardiovascular Sciences, Amsterdam UMC, University of Amsterdam, Amsterdam, Netherlands; ^2^ Department of Experimental Cardiology, Heart Center, Amsterdam Cardiovascular Sciences, Amsterdam UMC, University of Amsterdam, Amsterdam, Netherlands

**Keywords:** acetylcholine-activated potassium current, delayed afterdepolarizations, fast sodium current, *GNB5*, inward rectifier potassium current, *SCN5A*, triggered action potentials, ventricular action potential

## Abstract

Human-induced stem cell-derived cardiomyocytes (hiPSC-CMs) are a valuable tool for studying development, pharmacology, and (inherited) arrhythmias. Unfortunately, hiPSC-CMs are depolarized and spontaneously active, even the working cardiomyocyte subtypes such as atrial- and ventricular-like hiPSC-CMs, in contrast to the situation in the atria and ventricles of adult human hearts. Great efforts have been made, using many different strategies, to generate more mature, quiescent hiPSC-CMs with more close-to-physiological resting membrane potentials, but despite promising results, it is still difficult to obtain hiPSC-CMs with such properties. The dynamic clamp technique allows to inject a current with characteristics of the inward rectifier potassium current (I_K1_), computed in real time according to the actual membrane potential, into patch-clamped hiPSC-CMs during action potential measurements. This results in quiescent hiPSC-CMs with a close-to-physiological resting membrane potential. As a result, action potential measurements can be performed with normal ion channel availability, which is particularly important for the physiological functioning of the cardiac *SCN5A*-encoded fast sodium current (I_Na_). We performed *in vitro* and *in silico* experiments to assess the beneficial effects of the dynamic clamp technique in dissecting the functional consequences of the *SCN5A*-1795insD^+/−^ mutation. In two separate sets of patch-clamp experiments on control hiPSC-CMs and on hiPSC-CMs with mutations in *ACADVL* and *GNB5*, we assessed the value of dynamic clamp in detecting delayed afterdepolarizations and in investigating factors that modulate the resting membrane potential. We conclude that the dynamic clamp technique has highly beneficial effects in all of the aforementioned settings and should be widely used in patch-clamp studies on hiPSC-CMs while waiting for the ultimate fully mature hiPSC-CMs.

## 1 Introduction

Due to their immaturity, cardiomyocytes derived from human-induced pluripotent stem cells (hiPSC-CMs) are often depolarized and spontaneously active, largely due to an apparent lack of intrinsic inward rectifier potassium current (I_K1_). While maturation strategies are not yet common practice, the dynamic clamp is a relatively easy to set up technique to supply the hiPSC-CMs with a sufficient amount of synthetic I_K1_ to make them express a stable close-to-physiological resting membrane potential and exhibit a more cardiomyocyte-like action potential (AP). Starting with Bett et al. (2013), several labs and companies around the world have developed dynamic clamp systems to study hiPSC-CMs, each with their own preferences for hardware and the current-voltage relationship of the electronically expressed synthetic I_K1_ (see, e.g., Bett et al., 2013; Meijer van Putten et al., 2015; Kim et al., 2015; Goversen et al., 2018; Altomare et al., 2023). Yet, their common goal is to create quiescent hiPSC-CMs with a stable close-to-physiological resting membrane potential.

An alternative to the use of a dynamic clamp system to inject a synthetic I_K1_ is the straightforward injection of a hyperpolarizing current of fixed amplitude, as employed, for example, by Jara-Avaca et al. (2017). However, although such injection of a hyperpolarizing current of fixed amplitude, which obviously lacks a reversal potential, can produce a stable resting membrane potential near −80 mV in the absence of stimulation, the very same current can result in extremely negative diastolic potentials during 1 Hz stimulation (Verkerk et al., 2017). Furthermore, such an injection results in a dramatic shortening of the action potential due to the large additional outward current at plateau levels, resulting in quantitatively different drug effects (Verkerk et al., 2021b). Altogether, the action potential morphology becomes highly unsatisfactory (Verkerk et al., 2017) and the use of a dynamic clamp system is to be preferred.

These days, the dynamic clamp technique is routinely used in our patch-clamp laboratory. We have published several papers on the dynamic clamp technique *per se* (Wilders, 2006; Berecki et al., 2014; Verkerk et al., 2017; Verkerk and Wilders, 2021) as well as on studies in which the dynamic clamp technique was used as a tool in patch-clamp experiments on cultured or freshly isolated atrial cardiomyocytes (Majumder et al., 2020; Verkerk et al., 2021b) or on hiPSC-CMs (Meijer van Putten et al., 2015; Veerman et al., 2016; Portero et al., 2017; Veerman et al., 2017; Eroglu et al., 2020; Hilderink et al., 2020; Knottnerus et al., 2020; Verkerk et al., 2021a; Eroglu et al., 2021; Marchal et al., 2021; Koncz et al., 2022; Portero et al., 2022; Verkerk et al., 2022; Nasilli et al., 2023) to supply these cells with a synthetic I_K1_ during AP measurements.

In the present study, we re-analyzed experiments that were performed in order to optimize our current clamp phenotyping recordings for patch-clamp studies on control hiPSC-CMs and hiPSC-CMs with the 1795insD^+/−^ mutation in *SCN5A* (Veerman et al., 2016), the S81L^−/−^ mutation in *GNB5* (Verkerk et al., 2017; Veerman et al., 2019), and two distinct mutations in *ACADVL* (Knottnerus et al., 2020; Verkerk et al., 2021a). Also, we carried out computer simulations using a comprehensive model of a single ventricular-like hiPSC-CM in order to study the effects of the injection of a synthetic I_K1_ through dynamic clamp on individual membrane currents. Our results demonstrate how such an injection of a synthetic I_K1_ can make all the difference in patch-clamp studies on hiPSC-CMs.

## 2 Materials and methods

### 2.1 Generation, differentiation, and culturing of hiPSC-CMs

#### 2.1.1 Origin of hiPSC-CMs

In this study, we used hiPSC-CMs from cell lines that were generated for previous studies in which we participated by carrying out patch-clamp experiments on these hiPSC-CMs. To study the effects of the *SCN5A*-1795insD^+/−^ mutation, human induced pluripotent stem cell (hiPSC) lines were generated from a patient carrying the 1795insD mutation and from a healthy control, as detailed by Davis et al. (2012). To this end, skin biopsies were obtained after written informed consent of the individuals and approval by the medical ethics committees of the Leiden University Medical Center, Netherlands, and the Academic Medical Center, University of Amsterdam, Netherlands. To generate hiPSC lines for the study of the mutations in the *ACADVL* gene, fibroblasts from both patients and from a healthy control were reprogrammed to generate hiPSC lines, as set out by Knottnerus et al. (2020). These hiPSC lines were generated at the University Medical Center of the Georg August University of Göttingen, Germany, as approved by the local ethics committee and with the written consent of the individuals (Dudek et al., 2013). The homozygous S81L^−/−^ mutation in *GNB5* was inserted into a control hiPSC line using CRISPR/Cas9 technology, as described in detail by Veerman et al. (2019). This control hiPSC line was generated from the same healthy control as in the study of the mutations in the *ACADVL* gene, with approval from the local ethics committee and written consent from the healthy individual (Dudek et al., 2013).

#### 2.1.2 Differentiation and culturing of hiPSC-CMs

Details on the differentiation and culturing of the hiPSC-CMs that we used in our patch-clamp experiments are described elsewhere. The differentiation and culturing of the hiPSC-CMs used to study the effects of the *SCN5A*-1795insD^+/−^ mutation were as set out by Veerman et al. (2016). The differentiation and culturing of the hiPSC-CMs used to study the effects of the *GNB5*-S81L^−/−^ mutation were as described by Veerman et al. (2019), and atrial-like hiPSC-CMs were created by treatment with all-trans retinoic acid (RA) during the differentiation process, as described by Verkerk et al. (2017). Details on the differentiation and culturing of the hiPSC-CMs used to study the electrophysiological abnormalities due to deficiency in very long-chain acyl-CoA dehydrogenase (VLCAD) in response to mutations in the *ACADVL* gene are provided by Knottnerus et al. (2020) and Verkerk et al. (2021a).

### 2.2 Patch-clamp experiments

#### 2.2.1 Data acquisition

Recordings were made at 36°C ± 0.2°C using the perforated patch-clamp technique and an Axopatch 200B patch-clamp amplifier (Molecular Devices, Sunnyvale, CA, USA). Voltage control and data acquisition were realized with custom software (‘Scope’, version 04.04.27; kindly provided by J. G. Zegers). Data analysis was performed with custom software (“MacDAQ,” version 8.0; kindly provided by A. C. G. van Ginneken). Signals were low-pass filtered with a cut-off frequency of 5 kHz and digitized at 5 kHz for spontaneous AP recordings, at 40 kHz for stimulated APs, and at 3 kHz for delayed afterdepolarizations (DADs). Cell membrane capacitance (C_m_, in pF) was estimated by dividing the time constant of the decay of the capacitive transient in response to 5 mV hyperpolarizing voltage clamp steps from −40 mV by the series resistance, which was calculated from the membrane and access resistance analyzed during the 5 mV hyperpolarizing voltage step. Patch pipettes with a resistance of 2–3 MΩ were pulled from borosilicate glass (Harvard Apparatus, UK) and filled with a solution containing (in mM): 125 K-gluconate, 20 KCl, 5 NaCl, 0.44 amphotericin-B, 10 HEPES; pH adjusted to 7.2 (KOH). Cells were superfused with modified Tyrode’s solution containing (in mM): 140 NaCl, 5.4 KCl, 1.8 CaCl_2_, 1.0 MgCl_2_, 5.5 glucose, 5 HEPES; pH adjusted to 7.4 (NaOH). All potentials were corrected for the estimated liquid junction potential (Barry and Lynch, 1991) and any other offsets. In daily practice, this correction amounts to ≈−15 mV.

#### 2.2.2 Action potential recordings

We recorded both spontaneous APs and APs that were elicited at 1 Hz by overdrive stimulation with 3 ms, ≈1.2× threshold current pulses through the recording patch pipette. Susceptibility to DADs was tested by applying a 3 Hz pacing episode (10 s) followed by an 8 s pause. After the pause, a single AP was evoked to test the inducibility of early afterdepolarizations. DADs were defined as depolarizations larger than 1 mV that occurred after the fast pacing period. The AP parameters analyzed were maximum diastolic potential (MDP, in mV), maximum upstroke velocity ((dV_m_/dt)_max_, in V/s), AP amplitude (APA, in mV), AP amplitude at 20 ms after initiation of the upstroke (AP plateau, in mV), and AP duration at 20, 50, and 90% repolarization (APD_20_, APD_50_, and APD_90_, respectively, in ms). Parameters from 10 consecutive APs were averaged.

### 2.3 Dynamic clamp

#### 2.3.1 Dynamic clamp system

**FIGURE 1:**
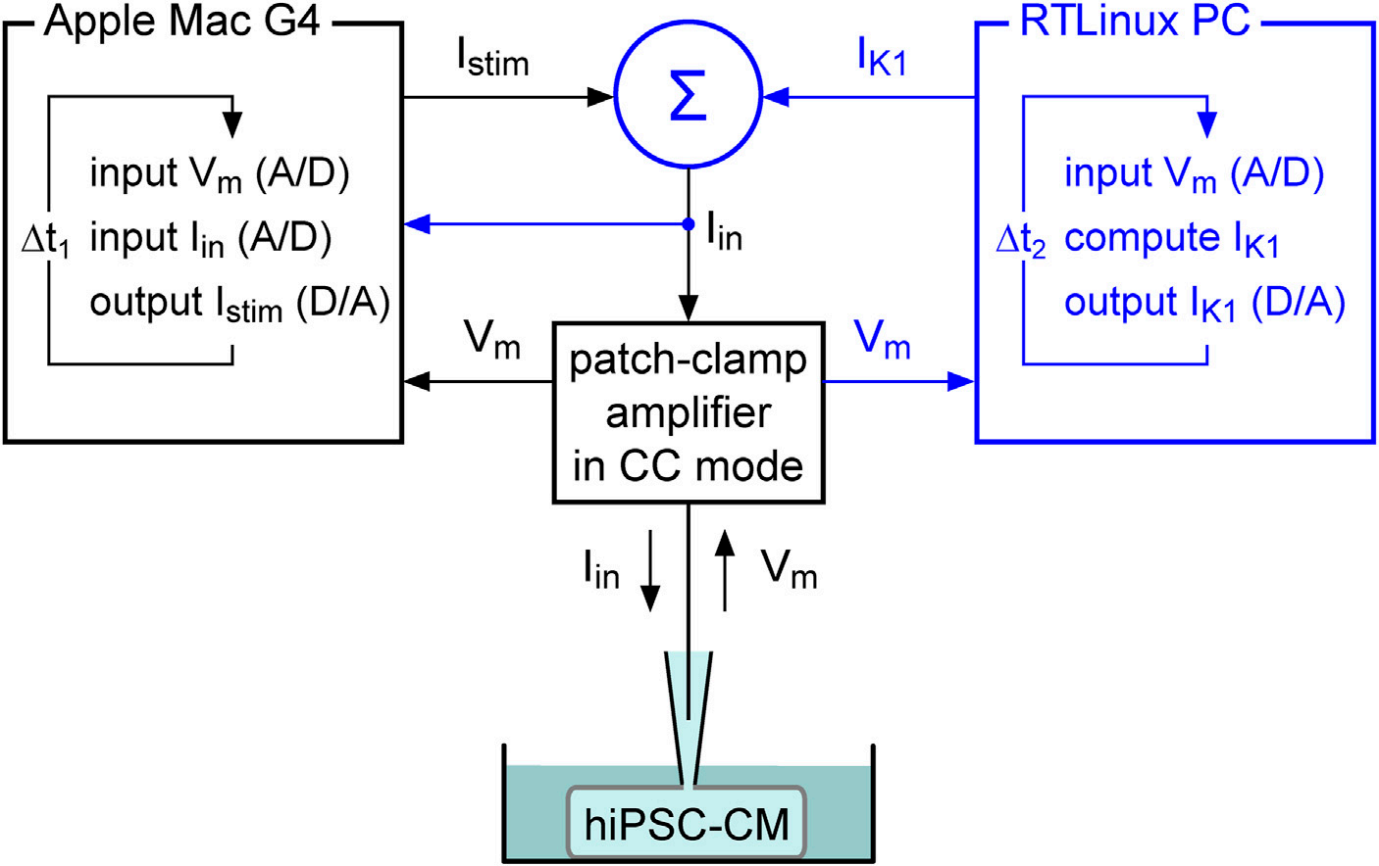
Dynamic clamp setup. The dynamic clamp component (represented by the blue section on the right) supplements the standard patch-clamp configuration (represented in black). The synthetic inward rectifier K^+^ current (I_K1_) is computed in real time by a Real-Time Linux (RTLinux) based PC in response to the recorded membrane potential (V_m_) and then added to any stimulus current (I_stim_). The resulting composite current (I_in_) is then transferred to the patch-clamp amplifier, which operates in current clamp (CC) mode and injects I_in_ into the patched hiPSC-CM. The I_K1_ and I_stim_ signals can be combined in a separate electronic box (Σ) or within the patch clamp amplifier itself, based on the amplifier input options. This process is updated with a time step Δt_2_. I_stim_ is sent out by the Apple Macintosh (Mac) G4 computer (or any other computer) that runs the regular patch-clamp software and is used to control the experiment. It records both V_m_ and I_in_ with a time step Δt_1_. Because I_in_ is read in, the latter computer contains all the data required for offline analysis, as in a regular patch clamp experiment.

In our dynamic clamp experiments, we extended our regular patch-clamp setup with a Real-Time Linux (RTLinux) based PC that continuously reads in the membrane potential (V_m_) of the patched hiPSC-CM (A/D). Figure 1 illustrates how this RTLinux based PC, in real time, computes the V_m_-dependent I_K1_ and sends out a command potential (D/A) that, after adding the command potential for any stimulus current I_stim_ (Σ), instructs the patch-clamp amplifier, which operates in current clamp mode (CC mode), to inject the resulting current I_in_ into the patched hiPSC-CM. Because I_in_ is continuously read in by the Apple Macintosh G4 computer that controls the experiment, the RTLinux based PC is only needed during the experiment and is not required for offline analysis of the acquired data.

#### 2.3.2 Injected I_K1_

To study the effects of an injected I_K1_ at a fixed stimulation frequency, we selected slowly beating hiPSC-CMs that we stimulated at an overdrive frequency of 1 Hz. The current density of the injected I_K1_ (expressed in pA/pF) was computed according to

$$I_{K1}=0.25955\times \left(V_{m}\;-\;E_{K}\right)\;/\;\left\{\;1+\exp\left[0.093633\times \left(V_{m}+72\right)\right]\right\},$$ (1)

where V_m_ denotes the membrane potential (expressed in mV and corrected for the liquid junction potential and any other offsets) and E_K_ denotes the potassium equilibrium potential (expressed in mV). E_K_ amounted to −86.9 mV in our experimental setting. According to Eq. 1, the injected I_K1_ exhibits a peak outward amplitude of 2 pA/pF, as routinely used in our laboratory (see, e.g., Veerman et al., 2016; Hilderink et al., 2020; Knottnerus et al., 2020; Verkerk et al., 2021a; Koncz et al., 2022; Verkerk et al., 2022). Such I_K1_ amplitude results in quiescent hiPSC-CMs with a resting membrane potential near −80 mV.

### 2.4 Computer simulations

#### 2.4.1 Simulating a single ventricular-like hiPSC-CM

The electrical activity of a single ventricular-like hiPSC-CM was simulated using the comprehensive model of such a cell that has been developed by Paci et al. (Paci et al., 2013; Paci et al., 2020) and is known as the Paci2020 model. We started from the CellML (Cuellar et al., 2003) code of the Paci2013 model (Paci et al., 2013), as publicly available from the CellML Model Repository (Lloyd et al., 2008) at https://www.cellml.org/ (accessed on 17 July 2017), which we updated to the Paci2020 model by applying the updates that were published from 2015 to 2020 (Paci et al., 2015; Paci et al., 2017; Paci et al., 2018; Paci et al., 2020). We carefully checked the resulting CellML code against the MATLAB code of the Paci2020 model that is publicly available from the MCBeng community of researchers in the field of Molecular and Cellular Bioengineering at https://www.mcbeng.it/en/ (accessed on 3 September 2023). The CellML code of the Paci2020 model was edited and run in version 0.9.31.1409 of the Windows-based Cellular Open Resource (COR) environment (Garny et al., 2003). All simulations were run for a period of 100 s, which was long enough to achieve stable behavior. The data analyzed are from the final 5 seconds of this 100 s period.

#### 2.4.2 Simulating the 1795insD mutation in *SCN5A*

To introduce the heterozygous 1795insD mutation in *SCN5A* (Proost et al., 2023) into the Paci2020 model, we split both the fast and the late sodium current (denoted by I_Na_ and I_NaL_, respectively) of the model cell into a wild-type and a mutant component, thus representing the state of these two related currents in a patient carrying the heterozygous 1795insD mutation. For the wild-type component we used the model’s regular I_Na_ and I_NaL_ equations with their fully-activated conductances (denoted by g_Na_ and g_NaL_, respectively) set to 50% of their control values. The experimentally observed −9.7 mV shift in the steady-state inactivation curve of the 1795insD mutant sodium current (Veldkamp et al., 2000) was applied to the inactivation equations of the mutant component. In their voltage clamp experiments, Veldkamp et al. (2003) found that the wild-type I_NaL_ was negligibly small, whereas the 1795insD mutant I_NaL_ was substantial, with an amplitude of ≈1.5% of the peak 1795insD mutant I_Na_ at potentials ranging from −20 to 0 mV. In line with these observations, we zeroed g_NaL_ when simulating the wild-type component of I_NaL_ and set g_NaL_ to 63.2 pS/pF when simulating the 1795insD mutant component of I_NaL_. The latter value of g_NaL_ was selected because it resulted in a 1795insD mutant I_NaL_ amplitude in our simulated voltage clamp experiments that amounted to ≈1.5% of the peak 1795insD I_Na_ at potentials ranging from −20 to 0 mV, consistent with the percentage observed *in vitro* by Veldkamp et al. (2003). The experimentally observed mutation-induced changes in the kinetics of inactivation and recovery from inactivation (Veldkamp et al., 2000; Viswanathan et al., 2001) were not taken into account.

### 2.5 Statistics

Data are expressed as mean ± SEM. Statistical analysis was performed using SigmaStat 3.5 software (Systat Software, Inc., San Jose, CA, USA). Two groups were compared with a paired or unpaired *t*-test, after testing the associated normality and equal variance assumptions with the Kolmogorov–Smirnov and Levene median tests, respectively. Three groups were compared by one-way ANOVA followed by a Holm–Sidak *post hoc* test. *p* < 0.05 was considered statistically significant.

## 3 Results

### 3.1 Current density and current-voltage relationship of I_K1_

#### 3.1.1 Current density and current-voltage relationship of I_K1_ in hiPSC-CMs

**FIGURE 2:**
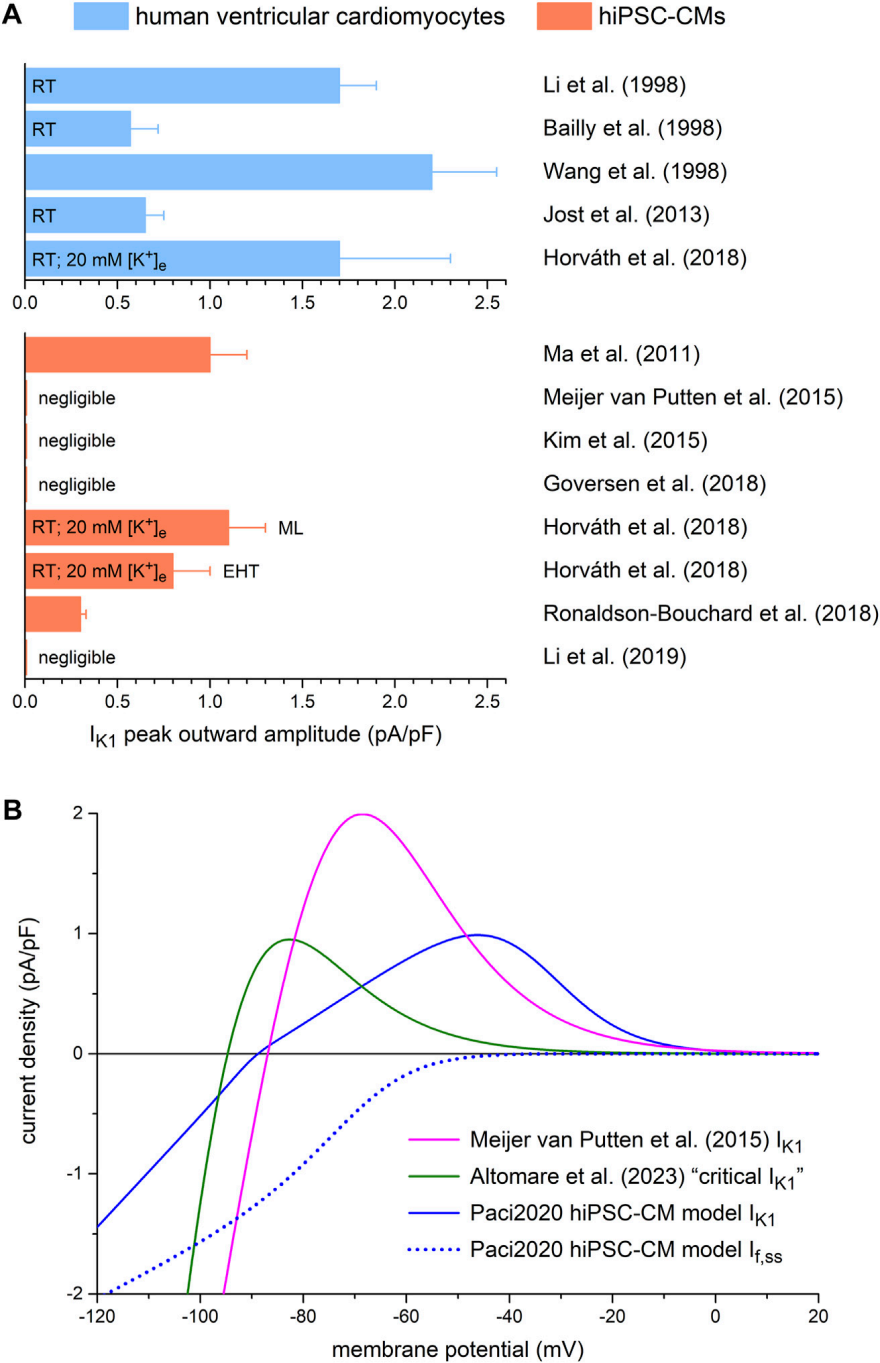
Amplitude of the inward rectifier K^+^ current (I_K1_) in isolated human ventricular cardiomyocytes and in hiPSC-CMs, and current-voltage relationship of I_K1_ used in dynamic clamp experiments. **(A)** Peak outward amplitude of I_K1_ in studies on isolated human ventricular cardiomyocytes (top) and in studies on hiPSC-CMs (bottom). RT: room temperature; [K^+^]_e_: extracellular K^+^ concentration; ML: cultured as monolayer; EHT: cultured as 3D engineered heart tissue. **(B)** Current-voltage relationships of I_K1_ used in the dynamic clamp studies of Meijer van Putten et al. (2015) and Altomare et al. (2023), and in the Paci2020 model of a ventricular-like hiPSC-CM (solid traces). The dotted trace shows the steady-state current-voltage relationship of the hyperpolarization-activated ‘funny current’ (I_f_) of the Paci2020 model.

Data on the amplitude of I_K1_ in human ventricular myocytes are relatively scarce and not unequivocal, as illustrated in Figure 2A (top panel). The average peak outward amplitudes reported by Bailly et al. (1998), Li et al. (1998), and Jost et al. (2013) range from 0.57 to 1.7 pA/pF. Of note, these values were all obtained at room temperature, which may explain why Wang et al. (1998), who carried out their experiments at 37°C, observed an average peak outward amplitude as high as 2.2 pA/pF. More recently, Horváth et al. (2018) observed an I_K1_ peak outward density of 1.7 ± 0.6 pA/pF (mean ± SEM, *n* = 13) in human left ventricular myocytes at room temperature. This is almost threefold larger than the value of 0.65 ± 0.1 pA/pF (mean ± SEM, *n* = 21) that was also observed in human left ventricular myocytes at room temperature by Jost et al. (2013). However, an important caveat is that Horváth et al. (2018) measured I_K1_ at an extracellular K^+^ concentration ([K^+^]_e_) of 20 mM (and a K^+^ concentration in the recording pipette of 120 mM), whereas it is known that the I_K1_ amplitude, including its peak outward amplitude, increases strongly with increasing [K^+^]_e_ (Bailly et al., 1998; O’Hara et al., 2011). Therefore, it is likely that the I_K1_ amplitude in the study by Horváth et al. (2018) is a significant overestimate of its physiological value.

The bottom panel of Figure 2A shows how these data on the amplitude of I_K1_ in human ventricular myocytes relate to data on the amplitude of I_K1_ observed in hiPSC-CMs. The latter data are even more divergent, although I_K1_ in hiPSC-CMs appears to be consistently smaller than in human ventricular myocytes. Ma et al. (2011) measured I_K1_ in their hiPSC-CMs as barium-sensitive current, as widely and successfully employed to measure I_K1_ in mammalian cardiomyocytes, and observed a substantial peak outward density of 1.0 ± 0.2 pA/pF (mean ± SEM, *n* = 6) at 37°C. However, this peak amplitude was achieved at a membrane potential of −35 mV, which is inconsistent with the value of −60 mV to −50 mV observed in human ventricular myocytes under close-to-physiological potassium concentrations (Bailly et al., 1998; Li et al., 1998; Wang et al., 1998; Jost et al., 2013). Subsequent attempts to measure I_K1_ as barium-sensitive current in hiPSC-CMs were often unsuccessful, suggesting that I_K1_ is small or even absent in hiPSC-CMs. For example, Meijer van Putten et al. (2015) observed an I_K1_-like barium-sensitive current in only 2 out of 7 cells, in either case with a negligible peak outward current. Similarly, Kim et al. (2015) found a negligible I_K1_ in their own hiPSC-CMs as well as in hiPSC-CMs prepared from the commercially available iCell hiPS cells (Cellular Dynamics International, Inc., Wisconsin, MI, USA). Goversen et al. (2018) found a barium-sensitive current with some similarity to I_K1_ in 7 out of 12 hiPSC-CMs. However, its current density was low, its reversal potential was less negative than expected for a potassium current, and its rectification was not as observed in adult cardiomyocytes. A barium-sensitive current “similar to I_K1_,” with characteristics highly similar to those observed by Goversen et al. (2018), was found by Li et al. (2019) in 4 out of 10 hiPSC-CMs.

On the other hand, Horváth et al. (2018) and Ronaldson-Bouchard et al. (2018) both observed a clearly non-negligible I_K1_ peak outward density in their hiPSC-CMs (Figure 2A, bottom panel). Ronaldson-Bouchard et al. (2018) reported an I_K1_ peak outward density of 0.30 ± 0.12 pA/pF at 37°C (mean ± SD, *n* = 18). However, their I_K1_ reached its peak outward amplitude at a membrane potential of ≈−40 mV, similar to the aforementioned value of −35 mV in the study by Ma et al. (2011), whereas the I_K1_ of human ventricular cardiomyocytes reaches its peak outward amplitude at −60 to −50 mV in the case of near-physiological potassium concentrations (Bailly et al., 1998; Li et al., 1998; Wang et al., 1998; Jost et al., 2013). Horváth et al. (2018) reported even larger I_K1_ peak outward densities of 1.1 ± 0.2 pA/pF (mean ± SEM, *n* = 67) and 0.8 ± 0.2 pA/pF (*n* = 56) at room temperature in hiPSC-CMs that were cultured as monolayer or as 3D engineered heart tissue, respectively. Yet, the application of a holding current in the range of 0.2 nA appeared necessary to elicit stable APs in most of their hiPSC-CMs. As in their experiments on human left ventricular myocytes, also performed at room temperature, Horváth et al. (2018) used a [K^+^]_e_ of 20 mM, which is known to significantly increase the peak outward I_K1_ amplitude, as set out above.

#### 3.1.2 Current density and current-voltage relationship of I_K1_ in dynamic clamp settings

One straightforward solution to compensate for the small or even absent I_K1_ in hiPSC-CMs (Figure 2A), and the consequent depolarized membrane potential and spontaneous activity, is to provide these cells with an I_K1_-like current through dynamic clamp. In our patch-clamp lab, we routinely inject the Meijer van Putten (2015) based I_K1_ of Figure 2B (magenta trace), with a peak outward amplitude of 2.00 pA/pF, into our hiPSC-CMs, as we also did in experiments of the present study. With this injected I_K1_ almost all of our hiPSC-CMs show a stable and hyperpolarized maximum diastolic potential (MDP) near −80 mV, not only the regular, ventricular-like ones (Table 1), but also the ones treated with all-trans retinoic acid (RA) during the differentiation process, as reviewed by Wiesinger et al. (2021), to increase the number of cells with atrial-like APs (Table 2). In our experiments, an injected I_K1_ with a smaller amplitude was not consistently successful, as was also observed by Altomare et al. (2023) when they injected the “I_K1__Ventr” of Figure 2B (green trace), with a peak outward amplitude of 0.99 pA/pF, into their hiPSC-CMs. In 17 out of 53 cells (32%), this “critical I_K1_” resulted in a prolonged or otherwise abnormal plateau phase, or it failed to hyperpolarize the cells to a close-to-physiological diastolic membrane potential (Altomare et al., 2023).

**TABLE 1:** Action potential parameters of control hiPSC-CMs in the absence and presence of 1 Hz overdrive stimulation and in the absence or presence of I_K1_ injection by dynamic clamp.

	No stimulation (spontaneously active) (*n* = 22)	1 Hz stimulation, no I_K1_ injection (*n* = 22)	1 Hz stimulation, I_K1_ injection (*n* = 32)
MDP (mV)	−69.4 ± 1.4	−66.8 ± 1.4	−82.1 ± 0.5
(dV_m_/dt)_max_ (V/s)	75.5 ± 16.7	58.8 ± 15.2	167.7 ± 19.9
APA (mV)	106.2 ± 2.7	93.8 ± 3.0	117.7 ± 1.5
AP plateau (mV)	104.0 ± 2.6	91.4 ± 3.3	114.0 ± 1.8
APD_20_ (ms)	84.5 ± 7.5	71.4 ± 7.2	89.8 ± 6.0
APD_50_ (ms)	134.2 ± 11.1	112.8 ± 11.0	149.0 ± 10.3
APD_90_ (ms)	170.5 ± 12.1	146.2 ± 12.7	179.0 ± 11.3
Cycle length (ms)	914.0 ± 112.4	N/A	N/A

Data are mean ± SEM. MDP, maximum diastolic potential; (dV_m_/dt)_max_, maximum upstroke velocity; APA, action potential amplitude; AP plateau, action potential plateau amplitude at 20 ms after initiation of the upstroke; APD_20_, APD_50_, and APD_90_, action potential duration at 20, 50, and 90% repolarization.

**TABLE 2:** Action potential parameters of RA-treated hiPSC-CMs in the absence and presence of 1 Hz overdrive stimulation and in the absence or presence of I_K1_ injection by dynamic clamp.

	No stimulation (spontaneously active) (*n* = 24)	1 Hz stimulation, no I_K1_ injection (*n* = 24)	1 Hz stimulation, I_K1_ injection (*n* = 31)
MDP (mV)	−69.4 ± 0.7	−71.7 ± 1.2	−82.1 ± 0.3
(dV_m_/dt)_max_ (V/s)	33.6 ± 5.5	58.3 ± 14.0	171.1 ± 18.4
APA (mV)	86.9 ± 3.6	82.3 ± 3.6	108.7 ± 3.8
AP plateau (mV)	78.5 ± 4.4	65.2 ± 4.9	80.2 ± 4.8
APD_20_ (ms)	40.9 ± 4.5	30.6 ± 4.4	32.4 ± 5.0
APD_50_ (ms)	74.8 ± 7.4	53.3 ± 8.6	57.3 ± 7.3
APD_90_ (ms)	128.8 ± 14.5	96.8 ± 8.1	81.1 ± 7.3
Cycle length (ms)	1078.7 ± 248.7	N/A	N/A

Data are mean ± SEM. MDP, maximum diastolic potential; (dV_m_/dt)_max_, maximum upstroke velocity; APA, action potential amplitude; AP plateau, action potential plateau amplitude at 20 ms after initiation of the upstroke; APD_20_, APD_50_, and APD_90_, action potential duration at 20, 50, and 90% repolarization.

Figure 2B (solid blue trace) also shows the current-voltage relationship of the I_K1_ that is included in the comprehensive model of a single ventricular-like hiPSC-CM that has been developed by Paci et al. (Paci et al., 2013; Paci et al., 2020) and is known as the Paci2020 model. This I_K1_ is largely based on the experimental data of Ma et al. (2011). Therefore, its peak outward amplitude is relatively large and obtained at a relatively depolarized membrane potential. For comparison, the dotted blue trace in Figure 2B shows the steady-state current-voltage relationship of the hyperpolarization-activated ‘funny’ current (I_f_) that is also included in the Paci2020 model. The amplitude of I_f_ in hiPSC-CMs is a matter of debate. In some studies (e.g., by Zhang et al. (2022)), it is a negligible current, whereas in others, e.g., in the studies by Ma et al. (2011) and Wang et al. (2021), it is quite prominent. In the latter studies, the steady-state amplitude of I_f_ at −80 mV is ≈1 pA/pF (*n* = 17) and ≈2 pA/pF (*n* = 14), respectively, comparable to or even larger than that in the Paci2020 model (Figure 2B). With such amplitudes, the injected synthetic I_K1_ is largely required to compensate for the substantial inward I_f_ near the MDP, which may explain, at least in part, why a relatively large I_K1_, as compared to the I_K1_ observed in human ventricular myocytes (Figure 2A, top panel), is necessary to obtain quiescent hiPSC-CMs with a stable and hyperpolarized resting membrane potential near −80 mV.

### 3.2 Dynamic clamp to obtain a fully functional fast sodium current in hiPSC-CMs

The availability of Na_V_1.5 sodium channels carrying the cardiac fast sodium current (I_Na_) is strongly dependent on cycle length and membrane potential. Consequently, I_Na_ in freshly isolated native CMs shows a lower amplitude at shorter cycle lengths, when there is less time available for recovery from inactivation between APs, and upon membrane depolarizations, when a larger fraction of I_Na_ channels is already inactivated at rest (Berecki et al., 2010). This is highly similar in hiPSC-CMs and as a result the maximum upstroke velocity ((dV_m_/dt)_max_) of the AP decreases significantly at high stimulus frequencies and depolarized membrane potentials (Verkerk and Wilders, 2021). Despite these well-known biophysical properties of I_Na_, studies on hiPSC-CMs still address this issue in different, suboptimal ways. Some studies just present AP parameters from depolarized, spontaneous APs, even when comparing APs obtained with an affected I_Na_ to control APs, as in the studies by El-Battrawy et al. (2018) and Kroncke et al. (2019). Under such conditions, I_Na_ is far from fully functional, and accordingly the results obtained may be disputable.

**FIGURE 3:**
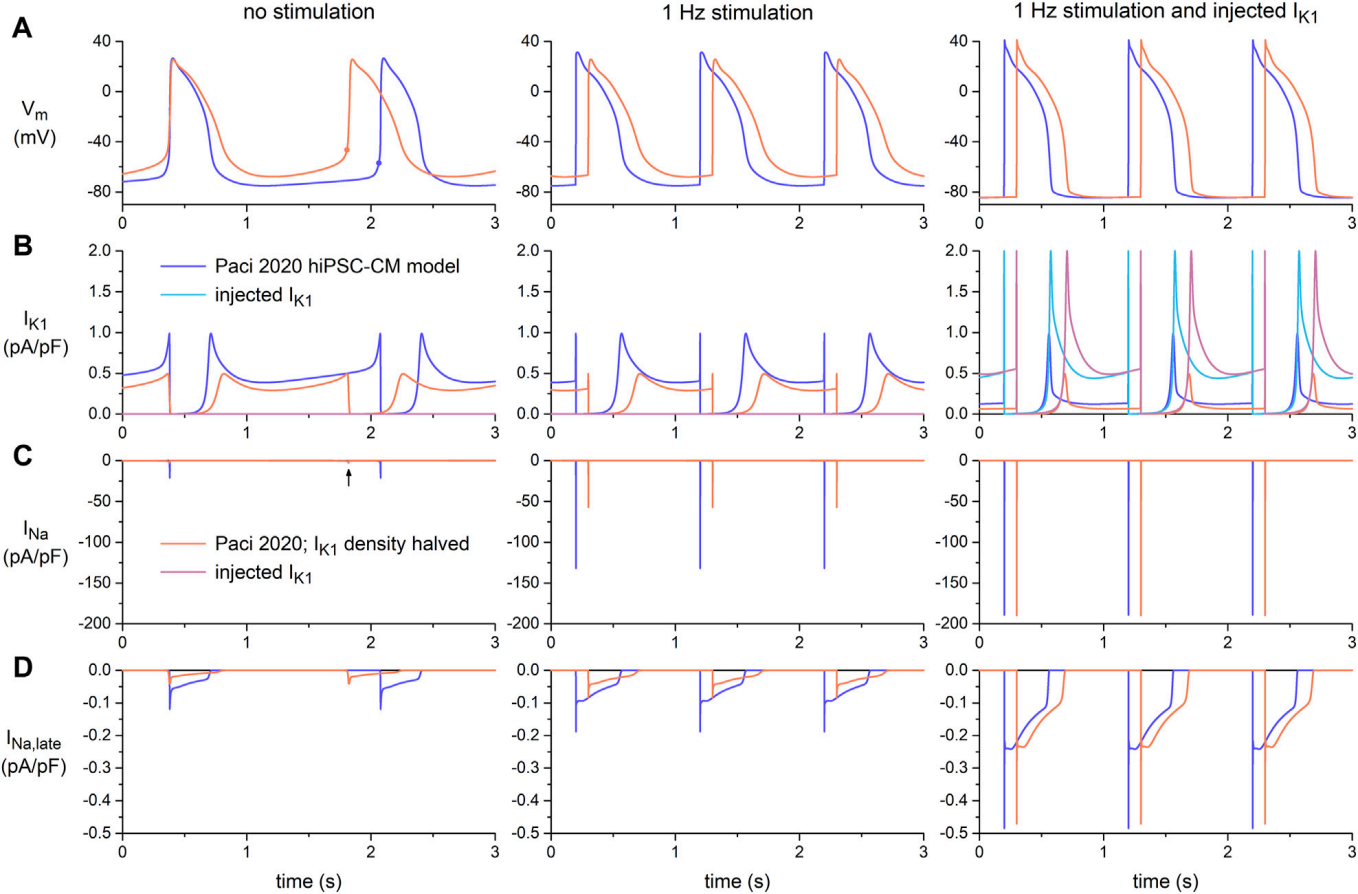
Electrical activity in the Paci2020 model of a ventricular-like hiPSC-CM during spontaneous activity (no stimulation; left panels), during 1 Hz stimulation (middle panels), and during 1 Hz stimulation combined with the simulated injection of the I_K1_ of Meijer van Putten et al. (2015) (right panels, reproduced with permission). The current-voltage relationship of this injected I_K1_ is shown in Figure 2B. The blue traces are obtained with the original native I_K1_ of the Paci2020 model and the orange ones with the current density of this native I_K1_ halved. **(A)** Membrane potential (V_m_). The filled circles in the left panel indicate the take-off potential (TOP) of the spontaneous action potentials. **(B)** I_K1_, with its native and injected components. **(C)** Fast sodium current (I_Na_). The vertical arrow in the left panel indicates the tiny I_Na_ observed when the simulated hiPSC-CM is spontaneously active and the current density of its native I_K1_ is halved. **(D)** Late sodium current (I_Na,late_).

In Figure 3, we illustrate how I_Na_ as well as the late sodium current (I_Na,late_) of the Paci2020 model of a single ventricular-like hiPSC-CM depend on the AP shape and the amount of native and injected I_K1_. In the left panels, there is no stimulation or injected I_K1_, and the simulated hiPSC-CM is spontaneously active at a rate of 35 beats/min (Figure 3A, left panel, blue trace). The native I_K1_ is rather large, with a peak outward amplitude of 0.99 pA/pF (Figure 2B, blue trace; Figure 3B, left panel, blue trace), and consequently the MDP is rather hyperpolarized with a value of −75.0 mV, similar to the value of −75.6 ± 1.2 mV (mean ± SEM, *n* = 32) observed by Ma et al. (2011) in their ventricular-like hiPSC-CMs, on which the Paci2020 model is largely based. However, in our own ventricular-like hiPSC-CMs, we observed an MDP of −69.4 ± 1.4 mV (mean ± SEM, *n* = 22; Table 1), and in another 15 studies on ventricular-like hiPSC-CMs, the mean MDP ranged from −39 to −72 mV, with most data between −55 and −65 mV (Verkerk and Wilders, 2021). The (dV_m_/dt)_max_ of the spontaneous AP amounts to 21.4 V/s and is largely determined by I_Na_, with a peak amplitude of 21.1 pA/pF (Figure 3C, left panel, blue trace). If we define the take-off potential (TOP) of these spontaneous APs as the membrane potential (V_m_) right before the AP at which its time derivative (i.e., dV_m_/dt) reaches a value of 0.5 V/s (Bucchi et al., 2007; Tóth et al., 2022), the TOP amounts to −57.0 mV, as indicated by the filled blue circle in Figure 3A (left panel). With an amplitude of ≈0.05 pA/pF during the plateau phase of the AP, I_Na,late_ is much smaller than the peak amplitude of I_Na_ (note the difference in ordinate scales between Figures 3C, D).

Because of the relatively large native I_K1_ and the associated relatively hyperpolarized MDP, we repeated our simulations with the current density of the Paci2020 model I_K1_ halved. The smaller I_K1_ (Figure 3B, left panel, orange trace) resulted in faster pacing, at a rate of 42 beats/min (Figure 3A, left panel, orange trace), a less negative MDP (of −67.9 mV, close the aforementioned value −69.4 ± 1.4 mV that we observed experimentally), and a less negative TOP (of −46.5 mV) (Figure 3A, left panel, filled orange circle). Because of the less negative membrane potential during the diastolic phase, I_Na_ is largely inactivated and its peak amplitude strongly reduced (to 2.6 pA/pF), so that it is barely visible in Figure 3C (left panel, orange trace), where its peak is indicated by a vertical arrow. The (dV_m_/dt)_max_ of the simulated AP is 5.4 V/s, which is reached during the second half of the slow upstroke and is largely determined by the L-type calcium current (not shown).

In the middle panels of Figure 3, the model cell is stimulated at a rate of 1 Hz, overdriving its spontaneous rate. Because the stimulus arrives early in the diastolic phase, the AP shows a more negative TOP than in the case of spontaneous activity. The TOP is −74.3 mV with the original Paci2020 model I_K1_ and −66.5 mV with its I_K1_ current density halved (Figure 3A, middle panel). These values are 17.4 and 20.0 mV more negative, respectively, than during spontaneous activity, so that much less I_Na_ is already inactivated at the onset of the AP. Accordingly, its peak amplitude during the upstroke is now substantially larger (132 and 57 pA/pF, respectively; Figure 3C, middle panel), which is associated with a substantially larger (dV_m_/dt)_max_ (132 and 67 V/s, respectively). Yet, the TOP still shows a difference of 7.8 mV, translating in a 57% decrease in I_Na_ peak amplitude and a 49% decrease in (dV_m_/dt)_max_, respectively, upon halving the current density of the model I_K1_, despite the identical conductance and kinetics of I_Na_.

In the right panels of Figure 3, the spontaneous activity of the model cell, either with its full native I_K1_ or with the current density of its native I_K1_ halved (blue and orange traces, respectively), was completely suppressed (Figure 3A, right panel) through the injection of a synthetic I_K1_ with a peak outward amplitude of 2 pA/pF (Figure 3B, right panel, magenta and light blue traces, respectively) and a current-voltage relationship as shown in Figure 2B (magenta trace). The associated TOPs are now nearly identical, with values of −84.3 and −84.1 mV, respectively, and similar to that of a ventricular myocyte (Verkerk and Wilders, 2021). Because of the nearly identical TOPs, the peak amplitude of I_Na_ (with a value of 189 pA/pF in both cases; Figure 3C) and the associated (dV_m_/dt)_max_ (with a value of 189 V/s in both cases) are now virtually identical, reflecting the identical conductance and kinetics of I_Na_. Similarly, I_Na,late_ is now largely identical (Figure 3D).

### 3.3 Dynamic clamp in dissecting the functional consequences of a mutation in *SCN5A*

**FIGURE 4:**
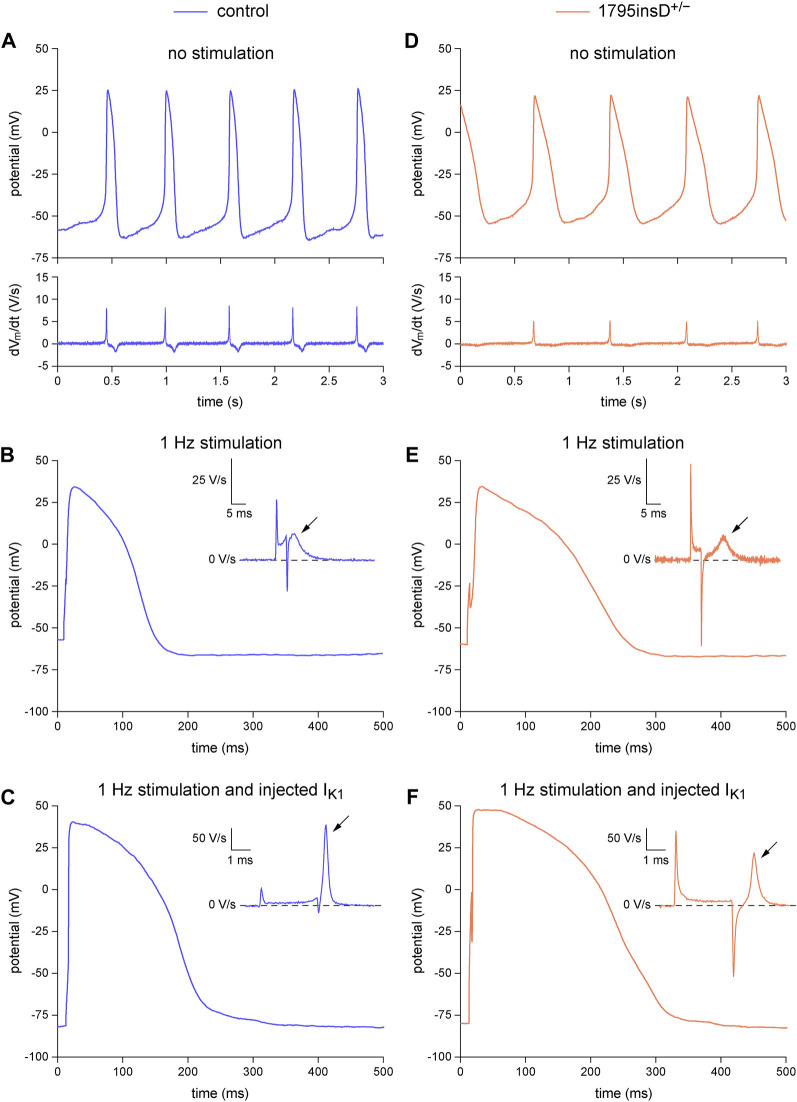
Dynamic clamp experiment to analyze the functional consequences of the heterozygous 1795insD mutation (1795insD^+/−^) in *SCN5A* in hiPSC-CMs. **(A–C)** Typical example of the action potentials (APs) from a control hiPSC-CM. **(A)** Train of spontaneous APs (top) and their time derivative (bottom). **(B)** Single AP obtained during 1 Hz stimulation and its time derivative near the AP upstroke (inset). **(C)** Single AP and its time derivative near the upstroke (inset) obtained during 1 Hz stimulation combined with the injection of a synthetic I_K1_. **(D–F)** Typical example of the APs from a 1795insD^+/−^ hiPSC-CM. **(D)** Train of spontaneous APs (top) and their time derivative (bottom). **(E)** Single AP obtained during 1 Hz stimulation and its time derivative near the AP upstroke (inset). **(F)** Single AP and its time derivative near the upstroke (inset) obtained during 1 Hz stimulation combined with the injection of a synthetic I_K1_. The slanted arrows in the insets indicate the maximum upstroke velocity immediately after the stimulus artefact.

In Figure 4 and the associated Table 3, we demonstrate the beneficial effects of the dynamic clamp technique in dissecting the functional consequences of the *SCN5A*-1795insD^+/−^ mutation in the pore-forming α-subunit of the I_Na_ channels, thus unveiling the phenotype of the mutation. In the absence of (overdrive) stimulation and synthetic I_K1_ injection, both types of hiPSC-CMs show spontaneous activity (Figures 4A, D, top panels) with an inevitably low (dV_m_/dt)_max_ (Figures 4A, D, bottom panels). Although the typical examples in Figures 4A, D, suggest an increase in AP duration and a decrease in I_Na_ (through the apparently lower (dV_m_/dt)_max_) as a result of the mutation, only the AP duration at 50% and 90% repolarization (APD_50_ and APD_90_, respectively) shows a statistically significant effect of the mutation (Table 3, left columns), despite the large number of hiPSC-CMs tested. The typical APs obtained with 1 Hz overdrive stimulation (Figures 4B, E) again suggest an increase in AP duration and a decrease in I_Na_ (through the apparently lower (dV_m_/dt)_max_; Figures 4B, E, insets). This is confirmed by the data collected in Table 3 (middle columns). The mutation still shows a statistically significant prolonging effect on the APD_50_ and APD_90_, but now also on the AP duration at 20% repolarization (APD_20_). Furthermore, the mutation has a statistically significant decreasing effect on (dV_m_/dt)_max_, suggesting a loss-of-function effect on the I_Na_ channels. Injection of a synthetic I_K1_ (with the characteristics of Figure 2B, magenta trace) into our hiPSC-CMs revealed further effects of the mutation. As suggested by the typical examples in Figures 4C, F, the mutation also affects the AP plateau. This was confirmed by the data collected in Table 3 (right columns). With the injected I_K1_, the mutation not only significantly lowers (dV_m_/dt)_max_ and significantly prolongs APD_20_, APD_50_, and APD_90_, as with 1 Hz overdrive stimulation *per se*, but also increases the AP amplitude (APA) as well as the AP amplitude at 20 ms after the onset of the upstroke (AP plateau), suggesting an increase in I_Na,late_ and thus a gain-of-function effect of the 1795insD^+/−^ mutation on I_Na,late_. These observations are consistent with the voltage clamp experiments on HEK-293 cells transfected with wild-type or 1795insD mutant cDNA by Veldkamp et al. (2000, 2003), who found a mutation-induced decrease in the amplitude of I_Na_ as well as a mutation-induced increase in the persistent component of I_Na_ (i.e., I_Na,late_).

**TABLE 3:** Action potential parameters of control and *SCN5A*-1795insD^+/−^ hiPSC-CMs in the absence and presence of 1 Hz overdrive stimulation and in the absence or presence of I_K1_ injection by dynamic clamp.

	No stimulation (spontaneously active)		1 Hz stimulation, no I_K1_ injection		1 Hz stimulation, I_K1_ injection	
	Control (*n* = 26)	1795insD^+/−^ (*n* = 28)	Control (*n* = 14)	1795insD^+/−^ (*n* = 16)	Control (*n* = 32)	1795insD^+/−^ (*n* = 23)
MDP (mV)	−61.2 ± 1.8	−57.3 ± 1.8	−69.6 ± 1.4	−69.3 ± 1.8	−81.3 ± 0.5	−80.8 ± 0.7
(dV_m_/dt)_max_ (V/s)	19 ± 5	12 ± 2	44 ± 6	18 ± 2***	187 ± 16	115 ± 13**
APA (mV)	84.7 ± 3.9	80.4 ± 3.0	96.7 ± 3.5	95.9 ± 2.6	117.3 ± 1.1	125.5 ± 1.6***
AP plateau (mV)	81.6 ± 3.7	78.3 ± 3.4	91.4 ± 3.5	93.8 ± 2.5	111.4 ± 1.8	118.0 ± 2.5*
APD_20_ (ms)	59.8 ± 4.4	79.3 ± 10.1	54.3 ± 5.2	89.3 ± 7.3***	80.4 ± 6.8	114.4 ± 8.6**
APD_50_ (ms)	99.4 ± 7.3	133.7 ± 13.8*	91.8 ± 8.9	153.1 ± 12.7***	143.4 ± 10.1	177.3 ± 13.0*
APD_90_ (ms)	153.4 ± 11.1	219.2 ± 20.3**	151.4 ± 15.1	233.1 ± 17.1**	194.3 ± 13.0	262.4 ± 16.7**

Data are mean ± SEM. MDP, maximum diastolic potential; (dV_m_/dt)_max_, maximum upstroke velocity; APA, action potential amplitude; AP plateau, action potential amplitude at 20 ms after initiation of the upstroke; APD_20_, APD_50_, and APD_90_, action potential duration at 20, 50, and 90% repolarization. *p < 0.05, 1795insD^+/−^ vs. control; **p < 0.01, 1795insD^+/−^ vs. control; ***p < 0.001, 1795insD^+/−^ vs. control.

**FIGURE 5:**
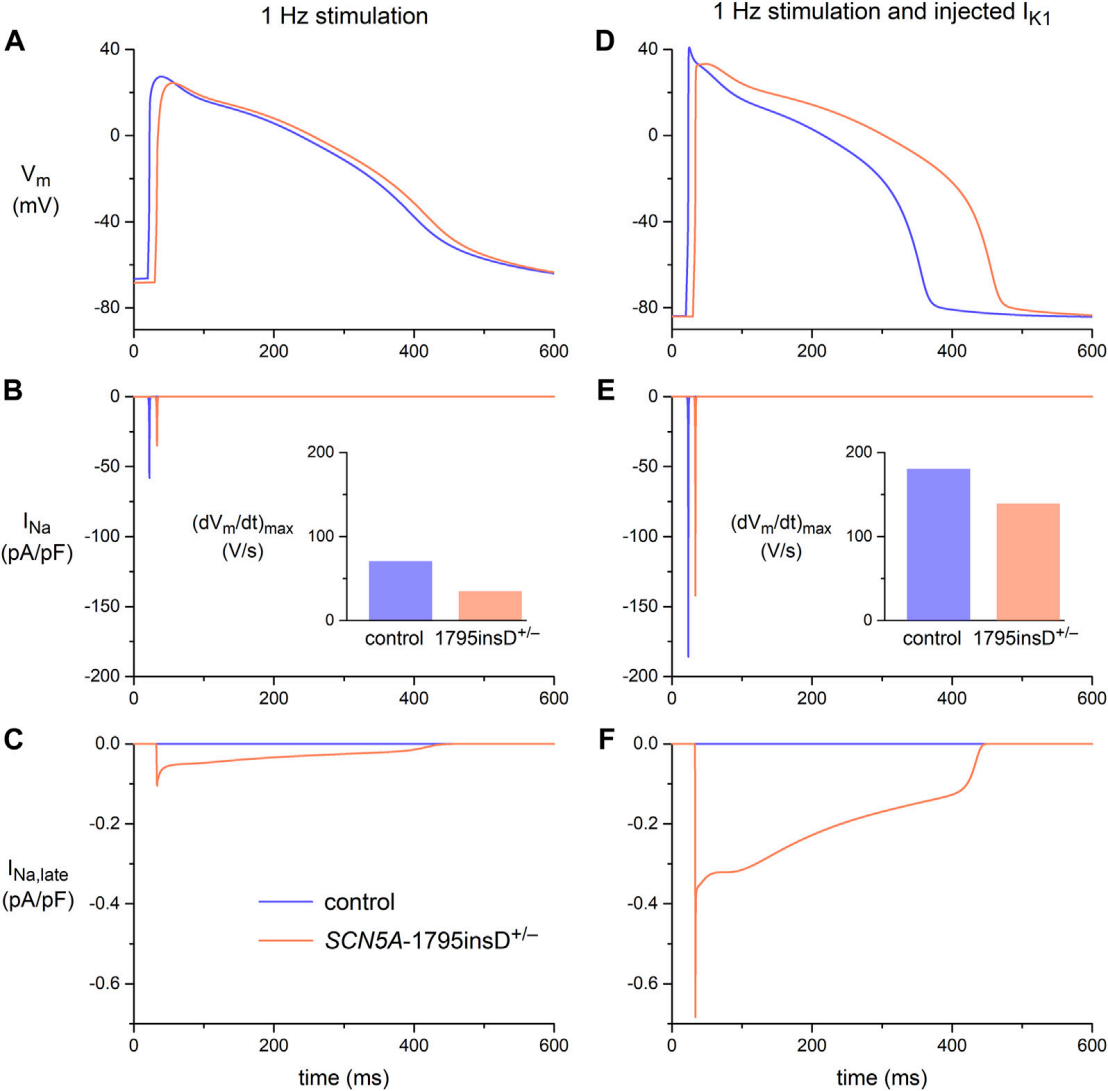
Membrane potential (V_m_), fast sodium current (I_Na_), and late sodium current (I_Na,late_) in the Paci2020 model of a ventricular-like hiPSC-CM under control conditions (blue traces) and when simulating the heterozygous 1795insD mutation (1795insD^+/−^) in *SCN5A* (orange traces). **(A)** V_m_, **(B)** I_Na_, and **(C)** I_Na,late_ during 1 Hz stimulation. **(D)** V_m_, **(E)** I_Na_, and **(F)** I_Na,late_ during 1 Hz stimulation combined with the simulated injection of a synthetic I_K1_ (Figure 2B, magenta trace). Data obtained with the current density of the native I_K1_ of the Paci2020 model halved. The insets to panels B and E show the maximum upstroke velocity ((dV_m_/dt)_max_).

The *in vitro* data in Figure 4 demonstrated that the effects of the 1795insD^+/−^ mutation in *SCN5A* on the persistent component of I_Na_ could only be revealed upon the injection of a synthetic I_K1_. *In silico* experiments with the Paci2020 model, with the current density of its native I_K1_ halved to better represent the experimental data from hiPSC-CMs, further emphasized the importance of the synthetic I_K1_ injection. The effects of the 1795insD mutation on I_Na_ and I_Na,late_, as observed in the voltage clamp experiments of Veldkamp et al. (2000, 2003), were introduced into the Paci2020 model as described in detail in Section 2.4.2. In short, to implement the 1795insD mutation, a −9.7 mV shift was applied to the steady-state inactivation curve of the mutant I_Na_ channels and the amplitude of I_Na,late_ was increased from zero under control conditions to ≈1.5% of the mutant peak I_Na_. With 1 Hz overdrive stimulation, but without injection of a synthetic I_K1_, there is no obvious difference between the AP obtained under control conditions and that in the case of the 1795insD^+/−^ mutation (Figure 5A). However, there is a mutation-induced decrease in I_Na_ (Figure 5B), which results in a significant decrease in (dV_m_/dt)_max_ (Figure 5B, inset). Apparently, the mutation-induced increase in I_Na,late_ (Figure 5C) is too small to have an immediate effect on the AP shape. However, upon injection of a synthetic I_K1_, both I_Na_ and I_Na,late_ are significantly increased (Figures 5E, F), due to the now close-to-physiological MDP. I_Na,late_ is now large enough to exert a clear effect on the AP shape, raising its plateau and increasing its duration (Figure 5D), as compared to control conditions. The increase in I_Na_ is associated with an increase in (dV_m_/dt)_max_, both under control conditions and in the presence of the 1795insD^+/−^ mutation (Figure 5E, inset), but the mutation-induced decrease in (dV_m_/dt)_max_ remains. Thus, our simulations also demonstrate that the injection of a synthetic I_K1_ is required to unveil the AP prolonging effects of the 1795insD^+/−^ mutation.

### 3.4 Dynamic clamp in detecting delayed afterdepolarizations

**FIGURE 6:**
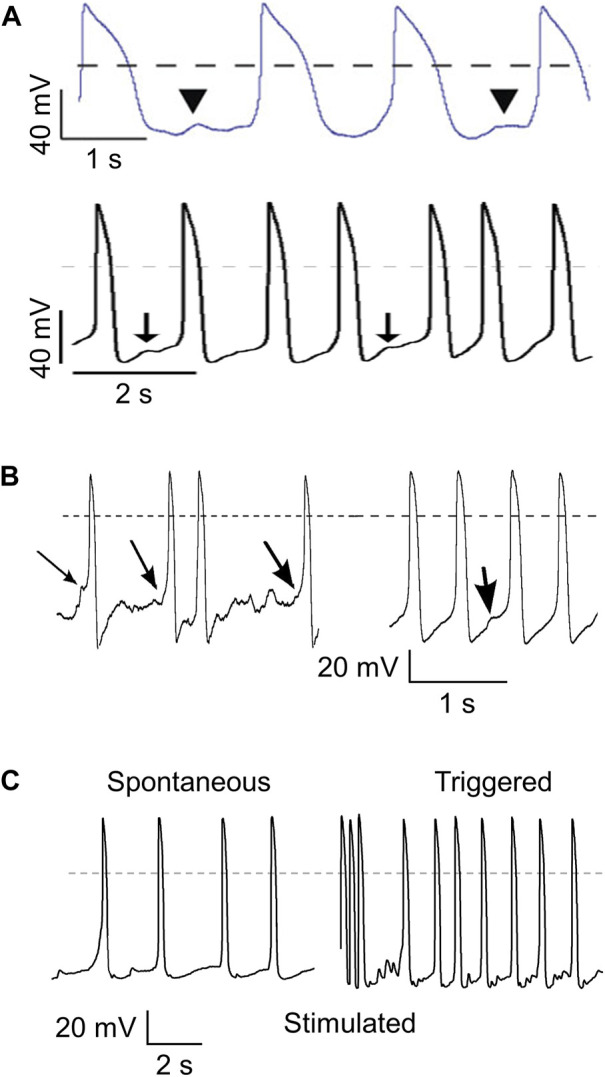
Different ways to detect delayed afterdepolarizations (DADs). **(A)** DADs defined as a subthreshold depolarization that interrupts a train of regular spontaneous action potentials (APs). Illustrations from the papers of Yazawa et al. (2011) (top; arrowheads indicating “putative DADs”. reproduced with permission) and Kujala et al. (2012) (bottom; arrows indicating “low-amplitude depolarizations that occur after the completion of repolarization, and have an amplitude of ≥3% of the preceding AP”. reproduced with permission). **(B)** DADs defined as an “abrupt depolarization” during “the typical “flat” period prior to initiation of an AP” (left; arrows indicating DADs) or as “spontaneous “DAD” behavior” during spontaneous pacemaker activity (right; arrow indicating DAD). Illustration taken from the paper by Kim et al. (2015), reproduced with permission. **(C)** DADs determined from the high frequency of “triggered” APs (right) during a 10 s pause following burst pacing (3 Hz, 10 s; final three stimulated APs shown) as compared to the relatively low frequency of spontaneous APs (left). Illustration taken from the paper by Devalla et al. (2016), reproduced with permission. Dashed lines indicate the 0 mV level.

Next, we assessed the value of dynamic clamp in detecting delayed afterdepolarizations (DADs). DADs are spontaneous depolarizations that occur after full repolarization of an AP and are due to spontaneous Ca^2+^ releases from the sarcoplasmic reticulum (SR) that activate the sodium-calcium exchanger in the cell membrane, resulting in a transient inward current (I_ti_) (Verkerk et al., 2000). DADs are common in calcium-overloaded cells and are associated with a number of inherited cardiac syndromes, such as catecholaminergic polymorphic ventricular tachycardia (CPVT) (Kujala et al., 2012; Devalla et al., 2016), Timothy Syndrome (Yazawa et al., 2011), hypertrophic cardiomyopathy (HCM) (Lan et al., 2013), and arrhythmias due to very long-chain acyl-CoA dehydrogenase deficiency (VLCADD) (Knottnerus et al., 2020; Verkerk et al., 2021a). HiPSC-CMs are increasingly used to study such syndromes, but the spontaneous activity of hiPSC-CMs may hamper the detection of DADs and/or their triggered APs. This is well illustrated by the different definitions of DADs and the different methods that have been used to detect these DADs in hiPSC-CMs studies. In some studies, a DAD is defined as the interruption of a train of regular spontaneous APs with a subthreshold depolarization (Yazawa et al., 2011; Kujala et al., 2012; Lan et al., 2013; Clemens et al., 2023), as illustrated in Figure 6A. Kim et al. (2015) described two types of so-called “DAD behavior”, i.e., (1) an abrupt depolarization after a period without depolarizations, and (2) a subthreshold depolarization between very regular spontaneous APs, as illustrated in Figure 6B, whereas Devalla et al. (2016) tested the susceptibility to DADs and their triggered APs by comparing spontaneous APs and the number of “triggered” APs after a fast pacing episode (3 Hz; 10 s), as illustrated in Figure 6C. Apart from the hindrance of the spontaneous APs to detect clear DADs, the amplitude of I_ti_ underlying the DAD is voltage dependent with a maximum amplitude around a membrane potential of −80 mV (Verkerk et al., 2000; Verkerk et al., 2001). Thus, it is conceivable that DADs in depolarized hiPSC-CMs have a lower amplitude, further limiting their detection.

**FIGURE 7:**
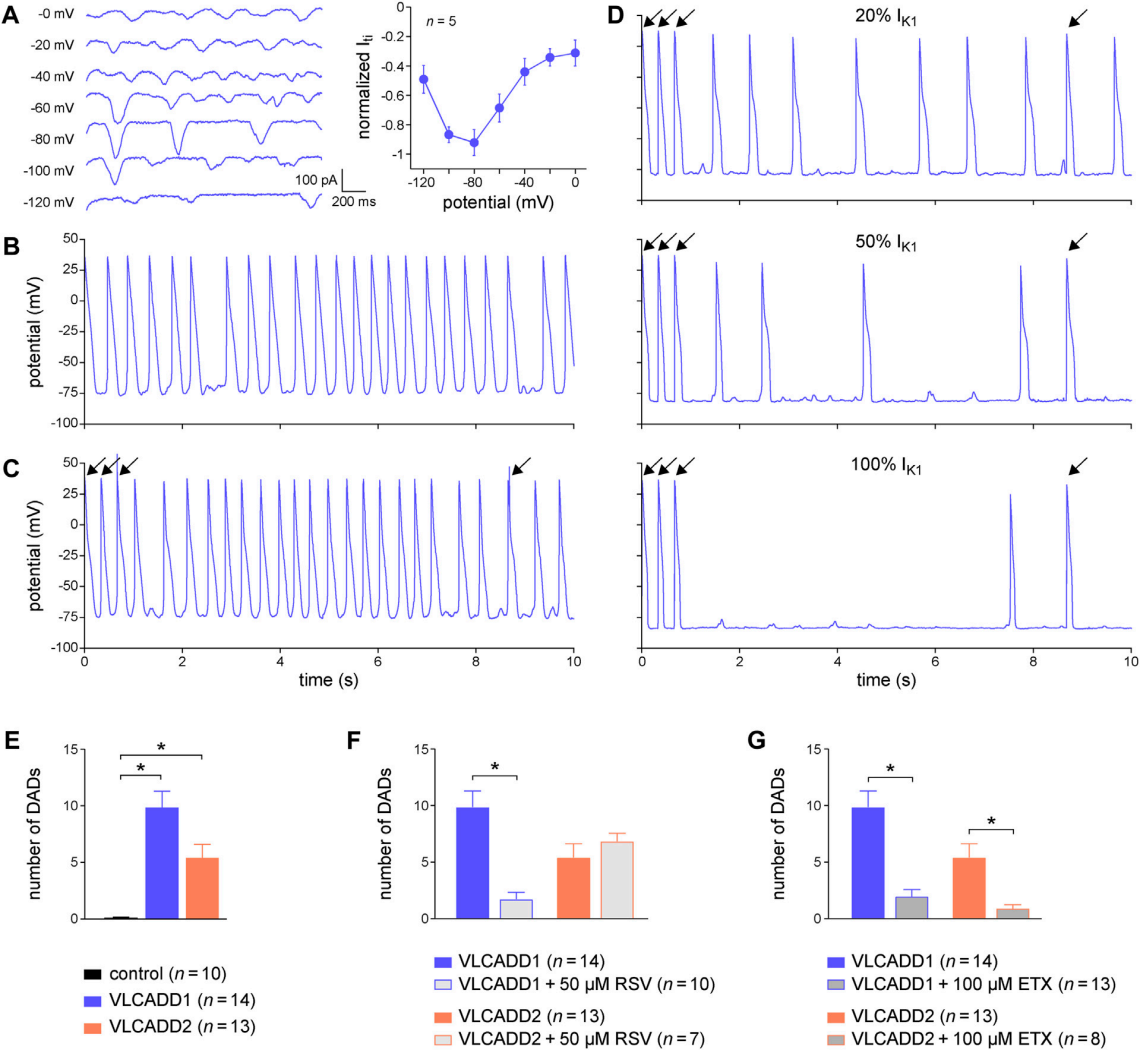
Dynamic clamp to facilitate the detection of delayed afterdepolarizations (DADs) in hiPSC-CMs. **(A)** Typical transient inward current (I_ti_) recordings in a single VLCADD1 hiPSC-CM at test membrane potentials ranging from −120 to 0 mV (left panel) and average I_ti_ amplitude versus membrane potential (right panel). **(B)** Typical train of spontaneous APs from a single VLCADD1 hiPSC-CM. **(C)** Current clamp recordings from a single VLCADD1 hiPSC-CM in the absence of the injection of a synthetic I_K1_ after a fast pacing protocol followed by an 8 s pause. Slanted arrows indicate APs elicited by a stimulus. **(D)** Current clamp recordings from a single VLCADD1 hiPSC-CM in the presence of the injection of a synthetic I_K1_ by dynamic clamp after a fast pacing protocol followed by an 8 s pause. The amount of I_K1_ injected varied between 20 (top panel), 50 (middle panel), and 100% (bottom) of the I_K1_ from Meijer van Putten et al. (2015) shown in Figure 2B. **(E)** Number of DADs (defined as >1 mV depolarizations) during the 8 s pause in control hiPSC-CMs and in hiPSC-CMs from the VLCADD1 and VLCADD2 lines. **(F)** Number of DADs during the 8 s pause in hiPSC-CMs from the VLCADD1 and VLCADD2 lines in the absence and presence of resveratrol (RSV; 50 µM). **(G)** Number of DADs during the 8 s pause in hiPSC-CMs from the VLCADD1 and VLCADD2 lines in the absence and presence of etomoxir (ETX; 100 µM). The number of DADs of each hiPSC-CM was determined from five 8 s episodes, each at a 100% injected I_K1_ amplitude. *p < 0.05.

Here, we tested the usefulness of dynamic clamp in detecting DADs in hiPSC-CMs generated from two patients who are homozygous or compound heterozygous for recessive mutations in the *ACADVL* gene that result in mitochondrial long-chain fatty acid oxidation (lcFAO) dysfunction and are associated with cardiac arrhythmias. The first patient, from whom the VLCADD1 hiPSC-CMs were generated, is compound heterozygous for missense mutations that generate a mitochondrial enzyme with residual activity, whereas the second patient, from whom the VLCADD2 hiPSC-CMs were generated, is homozygous for a truncating mutation that eliminates the enzyme activity. Both VLCADD1 and VLCADD2 hiPSC-CMs show higher systolic and diastolic intracellular Ca^2+^ concentrations than control hiPSC-CMs (Knottnerus et al., 2020). We first tested the voltage dependence of the I_ti_ in VLCADD1 hiPSC-CMs, as previously done in freshly isolated cardiomyocytes (Verkerk et al., 2000; Verkerk et al., 2001), by applying a fast pacing protocol consisting of a train of twenty 200 ms depolarizing pulses from −80 to 50 mV with a 100 ms interval, followed by a 3 s pause during which the membrane potential was clamped at test potentials ranging from −120 to 0 mV and I_ti_’s appeared. Figure 7A, left panel, shows typical I_ti_’s, while the average I_ti_ amplitude vs. potential relationship is depicted in Figure 7A, right panel. Similar to freshly isolated cardiomyocytes, I_ti_’s in hiPSC-CMs have their maximum amplitude around −80 mV. Figure 7B shows typical spontaneous APs from a VLCADD1 hiPSC-CM. At two points in time, near 2.5 and 9.0 s, spontaneous APs are interrupted and a subthreshold depolarization can be observed, but whether these are true DADs or failure of spontaneous APs is unknown. Figures 7C, D, shows current clamp recordings after a 10 s period of fast pacing (3 Hz) that is followed by an 8 s pause. Such a protocol is commonly used to detect DADs in freshly isolated cardiomyocytes (Den Ruijter et al., 2008). The final three APs evoked by stimulus pulses are indicated by an arrow. The recording in Figure 7C was made without I_K1_ injection and the number of APs observed was largely similar to that observed during regular spontaneous APs (Figure 7B). Figure 7D shows recordings obtained with I_K1_ injections of 20, 50, and 100% of our standard 2 pA/pF amplitude (i.e., 0.4, 1, and 2 pA/pF peak outward I_K1_). Obviously, the number of spontaneous APs decreases with increasing I_K1_ and the detection of DADs during the 8 s pause becomes easier as the amount of injected I_K1_ increases. Similar effects were found in another four hiPSC-CMs. We conclude that the detection of DADs is greatly enhanced by the use of dynamic clamp to inject a synthetic I_K1_ at the amplitude that we routinely apply in our patch-clamp experiments on hiPSC-CMs. Therefore, we also used such I_K1_ injection, i.e., with 100% of our standard 2 pA/pF amplitude, when assessing the number of DADs in both VLCADD cell lines under control conditions (Figure 7E), and when evaluating the effects of two compounds that affect lcFAO biochemistry, namely resveratrol (RSV) and etomoxir (ETX) (Knottnerus et al., 2020; Verkerk et al., 2021a). Interestingly, RSV (50 µM) only had a beneficial effect on VLCADD1 hiPSC-CMs (Figure 7F), whereas ETX (100 µM) had a beneficial effect on both VLCADD1 and VLCADD2 hiPSC-CMs (Figure 7G), rescuing the pro-arrhythmic phenotype with the many DADs. Recently, the usefulness of the dynamic clamp technique in detecting afterdepolarizations in hiPSC-CMs was further demonstrated by Portero et al. (2022), who injected a synthetic I_K1_ into hiPSC-CMs in a study of the effects of elevated branched-chain amino acid (BCAA) concentrations on the occurrence of afterdepolarizations.

### 3.5 Dynamic clamp in studies of factors modulating the resting membrane potential

**FIGURE 8:**
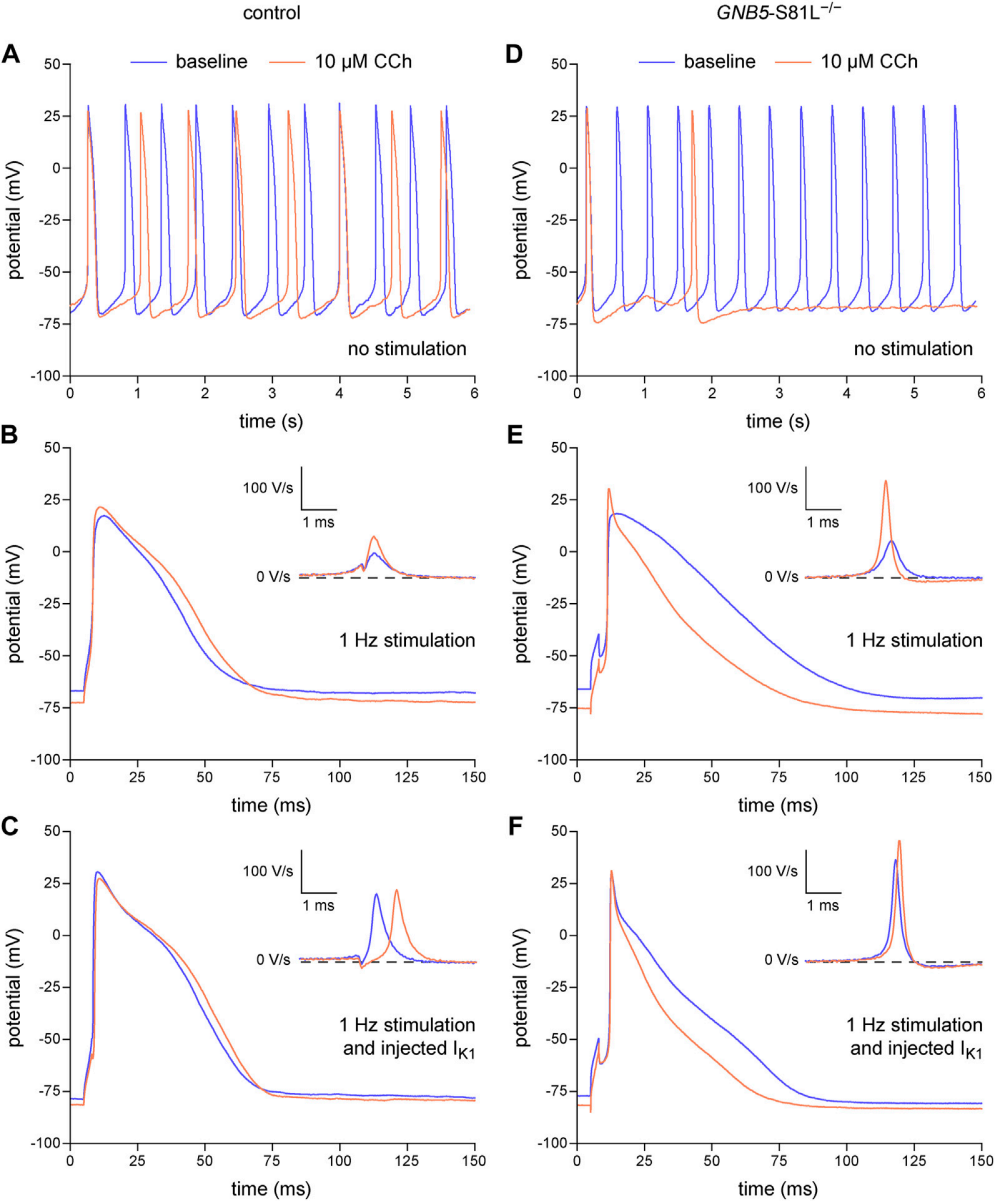
Dynamic clamp to study factors modulating the resting membrane potential of hiPSC-CMs. Effects of carbachol (CCh; 10 µM) on spontaneous APs, on APs during 1 Hz stimulation, and on APs during 1 Hz stimulation combined with the injection of a synthetic I_K1_ by means of dynamic clamp. **(A)** Typical spontaneous APs from a control atrial-like hiPSC-CM at baseline and after addition of CCh. **(B, C)** Typical APs from a control atrial-like hiPSC-CM **(B)** during 1 Hz stimulation and **(C)** from the same hiPSC-CM during 1 Hz stimulation combined with injection of I_K1_. **(D)** Typical spontaneous APs from an atrial-like hiPSC-CM carrying the homozygous S81L mutation (S81L^−/−^) in the *GNB5* gene. **(E,F)** Typical APs from an S81L^−/−^ atrial-like hiPSC-CM **(E)** during 1 Hz stimulation and **(F)** from the same hiPSC-CM during 1 Hz stimulation combined with injection of I_K1_. The insets show the time derivatives during the upstroke of the APs. The effects of CCh were determined after 4–5 min of application at the concentration of 10 µM. The current-voltage relationship of the injected I_K1_ is shown in Figure 2B (magenta trace).

In the above experiments, we have demonstrated that the dynamic clamp technique is a useful tool to study the electrophysiology of single hiPSC-CMs, but its application to inject a synthetic I_K1_ may in principle limit the detection of MDP modulating factors, because the MDP is importantly set by the synthetic I_K1_. However, we have recently shown in freshly isolated human atrial cardiomyocytes that the MDP can still be modulated by potassium current blocking agents such as apamin and barium when the MDP is pre-set at −80 mV (Verkerk et al., 2021b). Thus, as long as the MDP is not fully clamped at the E_K_, MDP changes may still be detectable. In a final set of experiments, we tested this hypothesis using the muscarinic receptor agonist carbachol (CCh; 10 µM) in atrial-like hiPSC-CMs injected with our standard synthetic I_K1_ with a peak outward amplitude of 2 pA/pF (Figure 2B, magenta trace). These experiments were not only carried out on control hiPSC-CMs, but also on hiPSC-CMs carrying the homozygous S81L^−/−^ mutation in *GNB5*. This gene encodes the G-protein β5 subunit (Gβ5), which has an inhibitory effect on the G-protein-coupled inward rectifier potassium (GIRK) channels that carry the ACh-activated potassium current (I_K,ACh_). Figures 8A–C, shows the effects of CCh in a control atrial-like hiPSC-CM that is spontaneously active (Figure 8A), stimulated at 1 Hz in the absence of the synthetic I_K1_ (Figure 8B), and stimulated at 1 Hz in the presence of the synthetic I_K1_ (Figure 8C). CCh induced a statistically significant hyperpolarization of the MDP, both in the absence and in the presence of the synthetic I_K1_, although the hyperpolarization was less pronounced in the presence of the synthetic I_K1_. Table 4 (left columns) summarizes the effects of CCh on all AP parameters, but does not include average data from spontaneous APs because spontaneous activity ceased in response to CCh in one out of the 10 cells studied. Similar effects were observed in an hiPSC-CM carrying the S81L^−/−^ mutation in *GNB5* (Figures 8D–F), but the CCh effects were more pronounced, with even cessation of spontaneous activity in 11 out of 14 cells, due to the increased density of the muscarinic receptor-activated K^+^ current (I_K,ACh_) (Veerman et al., 2019). Not only in the absence of the synthetic I_K1_ but also in its presence, CCh induced a statistically significant hyperpolarization of the MDP in the hiPSC-CMs with the *GNB5*-S81L^−/−^ mutation, and this hyperpolarization was larger in S81L^−/−^ hiPSC-CMs than in control hiPSC-CMs (Table 4, right columns). In conclusion, the MDP can still be modulated in the presence of a 2 pA/pF I_K1_ injection, and effects of inherited gene mutations can be observed.

**TABLE 4:** Action potential parameters of control and S81L^−/−^ hiPSC-CMs, stimulated at an overdrive frequency of 1 Hz, in the absence or presence of I_K1_ injection by dynamic clamp and in the absence or presence of 10 µM CCh.

			Control (*n* = 17)			S81L^−/−^ (*n* = 15)			
			Baseline	CCh	Difference	Baseline		CCh	Difference
Without I_K1_ injection									
	MDP (mV)		−69.8 ± 1.5	−72.5 ± 1.5***	−2.7 ± 0.5	−70.2 ± 1.8		−76.1 ± 1.7***	−5.9 ± 1.3^†††^
	(dV_m_/dt)_max_ (V/s)		65 ± 17	97 ± 22**	31.9 ± 10.2	59 ± 19		124 ± 28**	64.8 ± 34.8
	APA (mV)		83.4 ± 4.6	88.7 ± 4.4*	5.2 ± 2.4	88.6 ± 3.3		98.3 ± 3.4**	9.8 ± 5.1
	APD_20_ (ms)		28.7 ± 5.6	31.5 ± 6.1	2.8 ± 3.9	31.6 ± 2.4		23.1 ± 3.8**	−8.6 ± 4.9^†^
	APD_50_ (ms)		52.9 ± 8.6	53.7 ± 9.4	0.8 ± 3.3	57.2 ± 4.7		47.0 ± 6.2*	−10.3 ± 6.6^†^
	APD_90_ (ms)		104.7 ± 10.6	101.8 ± 11.3	−2.9 ± 4.1	90.4 ± 7.4		82.0 ± 10.1	−8.3 ± 7.4
With I_K1_ injection									
	MDP (mV)		−82.0 ± 0.4	−83.4 ± 0.4***	−1.4 ± 0.3	−82.2 ± 0.5		−84.6 ± 0.3***	−2.5 ± 0.4^†^
	(dV_m_/dt)_max_ (V/s)		215 ± 26	223 ± 25	8.4 ± 6.4	184 ± 32		199 ± 34	15.0 ± 8.0
	APA (mV)		108.7 ± 3.8	107.3 ± 3.8	−1.4 ± 1.2	109.4 ± 2.4		109.8 ± 2.6	0.4 ± 1.4
	APD_20_ (ms)		30.0 ± 8.3	28.9 ± 7.9	−1.1 ± 2.2	27.0 ± 4.4		23.1 ± 4.3*	−4.0 ± 1.8
	APD_50_ (ms)		57.7 ± 12.2	57.1 ± 12.4	−0.7 ± 3.0	62.2 ± 9.2		53.9 ± 12.1	−8.3 ± 4.6
	APD_90_ (ms)		85.7 ± 12.4	80.9 ± 13.0	−4.8 ± 3.2	86.1 ± 9.8		70.0 ± 12.0**	−16.1 ± 4.3^†^

Data are mean ± SEM. MDP, maximum diastolic potential; (dV_m_/dt)_max_, maximum upstroke velocity; APA, action potential amplitude; APD_20_, APD_50_, and APD_90_, action potential duration at 20, 50, and 90% repolarization. *p < 0.05, CCh vs. baseline; **p < 0.01, CCh vs. baseline; ***p < 0.001, CCh vs. baseline. ^†^ *p* < 0.05, S81L^−/−^ vs. control; ^†††^ *p* < 0.001, S81L^−/−^ vs. control.

## 4 Discussion

In the present study, we have demonstrated the benefits of injection of a synthetic I_K1_ through dynamic clamp in AP measurements and disease phenotyping of hiPSC-CMs with specific mutations in *SCN5A*, *GNB5*, and *ACADVL.* In short, the more close-to-physiological MDP due to the I_K1_ injection results in a more accurate determination of AP changes due to the *SCN5A*-1795insD^+/−^ mutation, and it also promotes the detection of DADs, while MDP can still be modulated. The strength of the injection of synthetic I_K1_ is not limited to the aforementioned specific gene mutations. It has also proven useful for studying drugs and compounds (Portero et al., 2017; Casini et al., 2019; Eroglu et al., 2020; Verkerk et al., 2021b; Eroglu et al., 2021; Nasilli et al., 2023), testing improvements in hiPSC-CM methodology (Giacomelli et al., 2020; Van den Brink et al., 2020), and studying other gene mutations. For example, *KCNH2* variants associated with a large loss of function of the rapid delayed rectifier current (I_Kr_) depolarized the MDP in single patch-clamped hiPSC-CMs to such an extent that APs could not be elicited (Van den Brink, 2022), but the AP phenotypes of these *KCNH2* mutations could still be determined in detail by the same group of researchers by the use of dynamic clamp (Brandão et al., 2020; Van den Brink et al., 2021). So far, dynamic clamp has also been successfully used to determine the AP phenotypes of mutations in *KCNA5* (Hilderink et al., 2020), *KCNJ2* (Meijer van Putten et al., 2015; Du et al., 2022), *KCNQ1* (Lee et al., 2021), and *CALM1* (Rocchetti et al., 2017), while we and others have also used the dynamic clamp in studies on the modulation of I_Na_ (Wang et al., 2022) and on mutations in *SCN5A* other than the above 1795insD (Veerman et al., 2017; Ma et al., 2018; Campostrini et al., 2023).

The usefulness of the dynamic clamp technique may depend on the selected current density and current-voltage (I-V) relationship of the injected I_K1_ (Meijer van Putten et al., 2015; Fabbri et al., 2019). For example, if the MDP is clamped at the E_K_ due to high densities of injected I_K1_, changes in MDP will be difficult to observe, if not completely absent. On the other hand, too low densities may result in too depolarized hiPSC-CMs, thereby severely reducing the I_Na_ availability, as shown in Section 3.2. In the present study, we used the I_K1_ of Figure 2B with a peak outward density of 2 pA/pF, which according to our experience is sufficient to clamp the MDP around −80 mV. In the study of Gaur et al. (2020), however, we had to increase the density to 4 pA/pF because the constant injection of a depolarizing current, used as a quantitative measure of repolarization reserve, otherwise resulted in spontaneous APs. As described before (Verkerk et al., 2021b), the required amount of current may also be influenced by the I_K1_ I-V relationship, the hiPSC-CM background, the differentiation and culturing methods, and the skill of the patch-clamper to minimize the ‘seal-leak current’, which is the current that flows due to the imperfect seal between the membrane and the patch pipette. Very recently, Clark et al. (2023) proposed to compensate for seal-leak currents during AP recordings from hiPSC-CMs. In our study, such an approach was not used. Although it is relatively easy to incorporate such compensation into dynamic clamp software, the determination and stability of this seal-leak current can be difficult, as discussed by Clark et al. (2023), and this might be even more evident when using the perforated patch-clamp technique (as we did), because some access by incorporation of amphotericin into the cell membrane occurs almost immediately after making seals.

In our studies, we normalized the injected I_K1_ to C_m_, which was determined based on the time constant of the decay of the transient capacitive current in response to a voltage clamp step (see Section 2.2.1). Other methods for determining C_m_ exist (Verkerk et al., 2000; Platzer and Zorn-Pauly, 2016), but the accuracy of the various methods for determining C_m_ was not compared in the present study. Therefore, potential small deviations introduced by different C_m_ determination methods must be taken into account when choosing the final injected I_K1_ density.

The I-V relationship of the injected I_K1_ was similar in our atrial- and ventricular-like hiPSC-CMs, whereas recently the use of an atrial-specific I_K1_ has been promoted for atrial-like hiPSC-CMs (Altomare et al., 2023). Treatment with all-trans retinoic acid (RA) during the differentiation process increases the percentage of hiPSC-CMs with atrial-like APs, but certainly not up to 100%, as also demonstrated by Altomare et al. (2023). Thus, both control and RA-treated hiPSC-CMs both form a mixed group of hiPSC-CMs, and there is no clear rationale for using different I-V relationships of the electronically expressed synthetic I_K1_ between groups. Nevertheless, we agree that the use of an atrial-specific I_K1_ may be useful for studying atrial-like hiPSC-CMs *per se*. Furthermore, the I-V relationships of the injected I_K1_ varied widely between labs (Section 3.1.2; Verkerk and Wilders, 2021). As long as the MDP is hyperpolarized to close-to-physiological values, this is not a real limitation, but researchers must realize that the choice of the I-V relationship may influence the outcome of studies, at least quantitatively. For example, in human atrial myocytes, we used two different I-V relationships, i.e., one with strong and one with moderate rectification (Verkerk et al., 2021b), and we observed that drug effects on the AP duration were quantitatively dependent on the selected I-V relationship. It is likely that this can be extrapolated to gene mutations that affect the AP duration.

We focused on the effects of injecting a synthetic I_K1_ in patch-clamp studies on hiPSC-CMs without paying much attention to the realization of the dynamic clamp extension of the patch-clamp setup, except for the global view in Figure 1. Yet, we would like to emphasize that, as an alternative to the RTLinux based extension shown in blue in Figure 1, one can make use of commercially available systems, such as the plug-and-play Cybercyte dynamic clamp systems from Cytocybernetics, Inc. (Buffalo, NY, USA) or the programmable data acquisition units with on-board microprocessor from Cambridge Electronic Design, Ltd. (Milton, Cambridge, England). Another commercial solution is to use a patch-clamp amplifier with built-in dynamic clamp options, such as the dPatch amplifier from Sutter Instrument (Novato, CA, USA). In our lab, we prefer to use an RTLinux based system (Dorval et al., 2001; Bauer et al., 2014), because of its high flexibility and relatively low cost. RTLinux is freely available from FSMLabs, Inc. (Austin, TX, USA). It runs in combination with the Linux operating system, which is also freely available. For the installation of the software, a not very modern, maybe already retired PC is sufficient. The most critical and most expensive part of the setup is the data acquisition board. However, it is not necessary to have a top-of-the-market high-speed board for the purpose of injecting a synthetic I_K1_. A mid-range board with 16-bit input and 16-bit output is sufficient, provided a Linux driver is available. In fact, a high-speed board usually achieves its high speed by buffering its input, which is of no use in our dynamic clamp setup because each individual A/D input must be processed in real time to generate its associated D/A output (Figure 1).

As already noted in Section 3.1.1, the experimental data on the peak outward amplitude of I_K1_ in human ventricular cardiomyocytes are quite variable (see also Figure 2A). Differences in recording temperature may explain some of the discrepancy. Based on the Q_10_ of 1.5 ± 0.3 (mean ± SD, *n* = 7) reported by Kiyosue et al. (1993), the peak outward amplitudes obtained at room temperature by Bailly et al. (1998) and by Jost et al. (2013) are not very different from that of Wang et al. (1998), but the difference with that observed by Li et al. (1998) persists. Slight differences in [K^+^]_e_ (either amounting to 4.0 or to 5.4 mM), apart from the large increasing effect when recording at a non-physiological [K^+^]_e_ of 20 mM, may also affect the I_K1_ amplitude, as may slight differences in K^+^ concentration in the recording pipette (ranging from 120 to 145 mM), affecting the reversal potential and driving force of I_K1_. Another factor affecting the observed peak outward amplitude is the exact concentration of Ba^2+^ used to identify I_K1_ as a barium-sensitive current, now that the block by Ba^2+^ may not be complete at the membrane potential of the peak outward I_K1_ (Bányász et al., 2007). A direct comparison of the peak outward I_K1_ amplitude of human ventricular cardiomyocytes and that of hiPSC-CMs is also obscured by such differences in recording conditions. However, Horváth et al. (2018) measured the peak outward I_K1_ amplitude of human left ventricular cardiomyocytes and that of hiPSC-CMs under identical recording conditions, at a [K^+^]_e_ of 20 mM, and observed a statistically significantly smaller peak outward amplitude of I_K1_ in their hiPSC-CMs.

Although we carried out our computer simulations with the Paci2020 model (Paci et al., 2020), several other models of a single hiPSC-CM are available, in particular those of Koivumäki et al. (2018), Kernik et al. (2019), and Akwaboah et al. (2021). However, our simulations mainly concern generic effects of changes in the activation and inactivation characteristics of the sodium current, which may differ quantitatively, but not qualitatively, between these models. Furthermore, the models by Kernik et al. (2019) and Akwaboah et al. (2021) show spontaneous beating rates of 62.0 beats/min (Kernik et al., 2019) and 126.0 beats/min (Akwaboah et al., 2021), respectively, so that these cannot be used in combination with the pacing rate of 1 Hz that we used in our *in vitro* and *in silico* experiments. The model developed by Koivumäki et al. (2018) shows a lower spontaneous beating rate than the other two models, amounting to 45.1 beats/min (Koivumäki et al., 2018), making it feasible as an alternative to the Paci2020 model that we used in our simulations. However, this model was constructed by merging the cell geometry and immature intracellular calcium handling of the mouse embryonic ventricular myocyte model of Korhonen et al. (2010) with the membrane electrophysiology of the hiPSC-CM model of Paci et al. (2015), using the ventricular-like variant of that model. Consequently, electrophysiological effects, in particular effects of changes in the sodium current, are not widely different between the Paci2020 model and the Koivumäki et al. (2018) model.

We defined the take-off potential (TOP) of the spontaneous APs of Figure 3A (left panel) as the membrane potential (V_m_) right before the AP at which its time derivative (dV_m_/dt) reaches a value of 0.5 V/s (Bucchi et al., 2007; Tóth et al., 2022). However, one should be aware that there is no universally accepted definition of the TOP of spontaneous APs and several other definitions have been used. For example, Honjo et al. (1996) determined the TOP from the intersection of a straight line fitted to the diastolic depolarization and a straight line fitted to the upstroke of their spontaneous APs. Somewhat similarly, Lyashkov et al. (2007) defined the TOP as the point of intersection of the AP at −20 mV projected down to the time axis and the extension of the straight line approximating the diastolic depolarization. Another definition was used by Larson et al. (2013), who defined the TOP as the membrane potential at which dV_m_/dt reaches 10% of its maximum value. Quite differently, Zaniboni et al. (2014) defined the TOP as the V_m_ at which its second time derivative (d^2^V_m_/dt^2^) reaches its maximum value. On the other hand, Kohajda et al. (2020) defined the TOP as the membrane potential at which d^2^V_m_/dt^2^ for the first time exceeds 15% of the maximum d^2^V_m_/dt^2^ in the time interval between the MDP and the overshoot potential. In computer simulations, where the MDP occurs at a sharp point in time, Xiong (2017) defined the TOP as the membrane potential at which dV_m_/dt equals the slope of the straight line connecting the MDP and the overshoot potential. In a computer simulation study with ‘noisy’ APs, we had used a similar definition, but with MDP +1 mV instead of MDP to overcome the indeterminate point in time at which the MDP occurs in case of a ‘noisy’ AP (Wilders and Jongsma, 1993). These different definitions, of which the above list is by no means exhaustive, may lead to quantitatively and perhaps also qualitatively different results, but a detailed analysis is beyond the scope of the present study.

The injection of a synthetic I_K1_ is a useful tool, but it is certainly not the ultimate solution to overcome the electrophysiological immaturity of hiPSC-CMs. Several approaches have been taken to create more mature hiPSC-CMs (Reilly et al., 2022; Yang et al., 2023). Maturation of hiPSC-CMs through long-term culturing results, among other advantages, in an increase of I_K1_ (Doss et al., 2012; Seibertz et al., 2023), although still not up to the level observed in human ventricular myocytes. On the other hand, the specific enhancement of I_K1_ through infection with adenoviral constructs of the *KCNJ2*-encoded I_K1_ channel protein Kir2.1 by Vaidyanathan et al. (2016) resulted in hiPSC-CMs with a peak outward current density of I_K1_ at −50 mV of 4.7 ± 1.5 pA/pF (mean ± SEM, *n* = 7), which is significantly higher than that observed in human ventricular myocytes (Figure 2A). Prolonged stimulation is another option. For example, Zhang et al. (2023) recently reported that the expression level of *KCNJ2* in hiPSC-CMs from Fujifilm Cellular Dynamics, Inc. (Wisconsin, WI, USA) was significantly increased by prolonged pacing.

## 5 Conclusion

Our results demonstrate how the injection of a synthetic I_K1_ through dynamic clamp can make all the difference in patch-clamp experiments on hiPSC-CMs. Because of these highly beneficial effects we conclude that the dynamic clamp technique should be widely used in patch-clamp studies on hiPSC-CMs while waiting for the ultimate fully mature hiPSC-CMs.

## Data Availability

The raw data supporting the conclusion of this article will be made available by the authors, without undue reservation.
